# Menstrual inequity in Spain: a cross-sectional study

**DOI:** 10.1093/eurpub/ckac129.307

**Published:** 2022-10-25

**Authors:** L Medina-Perucha, T López-Jiménez, C Jacques-Aviñó, AS Holst, C Valls-Llobet, J Munrós-Feliu, C Martínez-Bueno, D Pinzón-Sanabria, MM Vicente-Hernández, A Berenguera

**Affiliations:** 1 Institut de Recerca IDIAPJGol, Barcelona, Spain; 2 Universitat Autònoma de Barcelona, Barcelona, Spain; 3 Universitat Pompeu Fabra, Barcelona, Spain; 4 Centro de Análisis y Programas Sanitarios, Barcelona, Spain; 5 ASSIR, Institut Català de la Salut, Barcelona, Spain; 6 GRASSIR Research Group, Barcelona, Spain; 7 SomiArte Taller, Barcelona, Spain; 8 Universitat de Girona, Girona, Spain

## Abstract

**Background:**

Menstrual inequity has an impact on (menstrual) health outcomes and emotional wellbeing. It is also a significant barrier to achieve social and gender equity. The aim of this study was to assess menstrual inequity and its associations with sociodemographic factors, among women and people who menstruate (PWM) aged 18-55 in Spain.

**Methods:**

A cross-sectional online survey-based study was conducted in Spain in March-July 2021. Data were analysed through descriptive statistical analyses and multivariate logistic regression models.

**Results:**

22,823 women and PWM participated (Mean age=33.2, SD = 8.7). Over half had accessed healthcare services for menstruation (61.9%) and had partial/no menstrual education pre-menarche (58.4%). Lifetime menstrual poverty was 22.2-39.9%. Main risk factors for menstrual poverty were identifying as non-binary (aOR: 1.67, 95% CI, 1.32-2.11), being born in non-European or Latin American countries (aOR: 2.74, 95% CI, 1.77-4.24), and not having a permit to reside in Spain (aOR: 4.27, 95% CI, 1.94-9.38). In turn, menstrual poverty protective factors were having completed university education (aOR: 0.61, 95% CI, 0.44-0.84) and not experiencing financial hardship in the last 12 months (aOR: 0.06, 95% CI, 0.06-0.07). Besides, 75.2% of participants indicated having overused menstrual products because of not having access to adequate menstrual management facilities. Menstrual-related discrimination was reported by 44.0% of women and PWM. Menstrual discrimination risk was higher among non-binary menstruators (aOR: 1.88, 95% CI, 1.52-2.33). Menstrual-related work (20.3%) and educational (62.7%) absenteeism was reported.

**Conclusions:**

Our study suggests that menstrual inequity in Spain is widespread, especially among those more socioeconomically deprived, vulnerable migrant populations and non-binary and trans menstruators. Findings from this study are being useful to inform future research and menstrual (health) policies.

**Key messages:**

• Menstrual inequity especially impacts socioeconomically deprived, gender nonconforming menstruators and vulnerable migrant populations in Spain.

• Menstrual inequity research is crucial to address social inequities of health and develop menstrual policies.

